# Change of TGF-*β*1 Gene Expression and TGF-*β*1 Protein Level in Gingival Crevicular Fluid and Identification of Plaque Bacteria in a Patient with Recurrent Localized Gingival Enlargement before and after Gingivectomy

**DOI:** 10.1155/2018/3670583

**Published:** 2018-08-05

**Authors:** Lilies Anggarwati Astuti, Mochammad Hatta, Sri Oktawati, Rosdiana Natzir, Ressy Dwiyanti

**Affiliations:** ^1^Post-Graduate Program of Medical Sciences, Faculty of Medicine, University of Hasanuddin, Makassar, Indonesia; ^2^Molecular Biology and Immunology Laboratory, Faculty of Medicine, University of Hasanuddin, Makassar, Indonesia; ^3^Department of Periodontology, Faculty of Dentistry, University of Hasanuddin, Makassar, Indonesia; ^4^Department of Biochemistry, Faculty of Medicine, University of Hasanuddin, Makassar, Indonesia; ^5^Department of Medical Microbiology, Faculty of Medicine, Tadulako University, Palu, Indonesia

## Abstract

This case report highlights the change of TGF-*β*1 gene expressions and TGF-*β*1 protein level in gingival crevicular fluid (GCF) and identification of plaque bacteria in a patient with recurrent localized gingival enlargement before and after *gingivectomy* treatment. A 26-year-old woman came to AG Dental Care Clinic, South Sulawesi, Indonesia, in October 2015 with a chief complaint that her gingiva often bled spontaneously and she felt pain on her gingiva and felt less comfortable and no self-confidence with her anterior and posterior gingival condition on the right maxilla region which is slightly larger than normal. She often felt that her gingiva could bleed spontaneously when she was talking or remains silent though. The patient is disturbed by the malodor she felt. At that moment, the patient sought for gingivectomy treatment. Three years afterward, the patient came back with the same complaint. Gingival crevicular fluid has been taken from the gingival sulcus before and after gingivectomy. Clinical and GCF follow-up examination was performed one week and three weeks after gingivectomy, and successful results on biological, functional, and aesthetic parameters were observed.

## 1. Introduction

The gingival disease has common features such as an increase in the size of the gingiva. This condition nowadays is known as gingival enlargement or gingival overgrowth [1]. This term has replaced gingival hyperplasia (increase in cell number) and gingival hypertrophy (increase in cell size) as these are histological diagnosis and do not accurately describe the varied pathological processes seen within the tissues [2].

Gingival enlargement or gingival overgrowth is a common finding in clinical practice. This condition affects the patient's oral hygiene practice and aesthetics and hampers speech, mastication, and natural self-confidence [3]. Many types of gingival enlargement can be classified according to etiologic factors and pathologic changes such as inflammation, drug-induced enlargement, systemic diseases or conditions, neoplasms, and false enlargement. Gingival enlargement can be designated using the criteria of location and distribution along with the degree [1].

Based on distribution, gingival enlargement may be described as localized or generalized [2, 4]. Localized gingival enlargements are limited to the gingiva adjacent to a single tooth or a group of teeth that started as ballooning papillae and then progressed to involve the marginal gingiva and in more severe cases can cover occlusal aspects of dentition [1, 2]. Historically, this condition termed as epulis refers to any solitary/discrete, pedunculated, or sessile masses of the gingiva with no histologic characterization of a particular lesion. The precise term “reactive lesion of the gingiva” seems to be more appropriate for these swelling conditions [2, 4].

Gingival enlargement commonly was an inflammatory process related to plaque accumulation and trauma. This condition has the clinical appearance such as soft, edematous, hyperemic or cyanotic, and usually painful or at least sensitive. These gingivae bleed quickly when probed and have smooth and distended appearance; the normal stippling has usually been lost clinically as well [2, 5].

The appropriate treatment for gingival enlargement depends on precisely diagnosing the cause of enlargement. Gingival enlargement caused by plaque (inflammatory enlargement) should be resolved with nonsurgical treatment including debridement of plaque and calculus (scaling and root planing), improved oral hygiene (oral hygiene instruction), and administration of antibiotics, usually amoxicillin and metronidazole, along with anti-inflammatory (ibuprofen) and analgesic (paracetamol) drugs and the use of chlorhexidine mouth rinse [5, 6].

If the resolution of enlargement did not occur, resulting in the persistence of periodontal pocket such as in fibrotic gingival tissues, this condition may require more detailed assessment and a longer-term management plan. Surgical management to remove enlarged tissue such as the use of laser/electrosurgery excision and internal/external bevel gingivectomy can be provided to improve access for the patient's oral hygiene [5, 6]. Removal must be thorough and based on the understanding of the lesion type. This removal usually includes complete excision of the lesion after the elevation of full-thickness mucoperiosteal flaps and thorough curettage of the area to its origin from the periosteum and periodontal ligament cells to prevent recurrence. Sutures were then given after achieving proper hemostasis [7–9].

Hence, plaque control is an essential aspect of management in all patients. An excisional/incisional biopsy and/or hematologic/histologic examination may be needed occasionally to precisely diagnose the uncommon cases of gingival enlargement. Every effort should be made to obtain primary closure of the surgical site to facilitate healing and so discourage the proliferation of granulation tissue which heralds early recurrence. A follow-up is required to ensure that any recurrence is detected early and dealt with and that the postsurgical gingival contour is maintained as close as possible to its preoperative state [4, 7].

## 2. Case Presentation

A 26-year-old woman came to AG Dental Care Clinic, South Sulawesi, Indonesia, in October 2015 with a chief complaint that her gingiva often bled spontaneously and she felt pain on her gingiva and felt less comfortable and no self-confidence with her anterior and posterior gingival condition on the right maxilla region which is slightly larger than normal. She often felt that her gingiva could bleed spontaneously when she was talking or remains silent though. The patient is disturbed by the malodor she felt. At that moment, the patient sought for gingivectomy treatment. Three years afterward, the patient came back with the same complaint. Gingival crevicular fluid has been taken from the gingival sulcus before and after gingivectomy. Clinical and GCF follow-up examination was performed one week and three weeks after gingivectomy, and successful results on biological, functional, and aesthetic parameters were observed.

The expected results with the gingivectomy treatment are that patients should not perceive any more complaint such as spontaneously gingival bleeding, pain on the gingiva, and malodor. Besides, after the gingivectomy treatment, the patient already felt comfortable and had her self-confidence back with her anterior and posterior gingival condition on the right maxilla region not having the appearance that is slightly larger than normal. Besides, the expected results after gingivectomy and scaling and root planing treatment such as localized gingival enlargement on the anterior and posterior of the right maxilla region do not recur.

Gingival crevicular fluid (GCF) was taken from the gingival area with enlargement using a paper point. The paper point was inserted into the gingival sulcus to absorb the gingival fluid. Then, the paper point was inserted to medium fluid L6. GCF was then checked using real-time polymerase chain reaction (RT-PCR) to find TGF-*β*1 gene expression and examined using enzyme-linked immunosorbent assay (ELISA) to find TGF-*β*1 protein level (Table 1). On the other hand, smear plaque was taken from the tooth surface both supragingival and subgingival and then inserted to medium transport.

**Figure 1:**
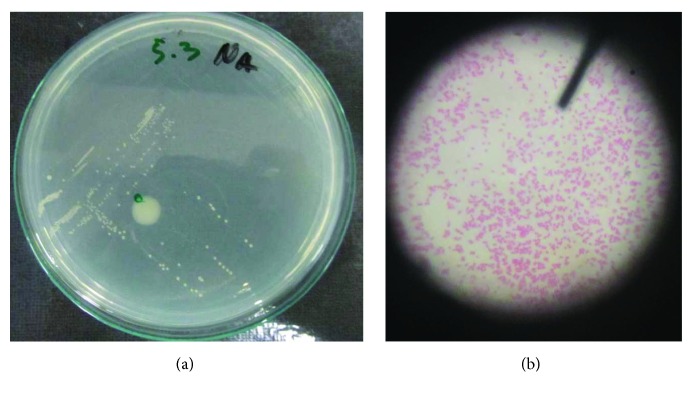
The smear of dental plaque on the medium to show the growth of *Streptococcus* sp. bacteria on the control at day 21 (a). Under a microscope with 1000x magnification, the growth of *Klebsiella* sp. bacteria was visible at the time before gingivectomy and at the time of the first control at day 7 (b).

Furthermore, the transport medium containing plaque and calculus was taken to the microbiology laboratory for bacterial culture examination, and the bacterial culture was cultured using Brain-Heart Infusion Broth (BHIB) medium (Figure 1). Then, the observation of swabs of dental plaque samples incubated for 1 × 24 hours in the incubator at a certain temperature was conducted. Identification of bacteria under a microscope was performed to determine bacterial species based on bacterial morphology before and after gingivectomy treatment (Table 2). On excised gingival tissue, biopsy was performed to find tissue morphology and tumour subtype and to grade gingival cells.

## 3. Discussion

Periodontal disease is multifactorial, including the case with recurrent localized gingival enlargement. When microbial (bacterial biofilm) and other environmental factors such as gender were believed to initiate and modulate periodontal disease development, now there has been powerful supporting data explaining that genetic and environmental factor risk plays a role in the trend for recurrence and severity development of periodontal disease. The enlargement could be due to a reduction of collagen degradation by collagenase or the outcome of overproduction of extracellular ground substance. Some literature has reported the synergistic effect of proinflammatory cytokines on the possible factors involved in this enlargement [10]. Genetic and technology information applied for prediction, diagnosis, and periodontal condition treatment conceptually is very interesting at this moment. Some features such as cytokines, cell surface receptors, chemokines, and enzymes, related to the recognition of antigen, immune system, and host response, among the other things, are determined by a polymorphism genetic component that possibly increases the individual's vulnerability to periodontal disease. Growth factors and cytokines play an important role in the regulation of the gingival extracellular matrix turnover. Tumour necrosis factor-*α* (TNF-*α*) and interleukins induce the expression of MMPs while transforming growth factor-*β* (TGF-*β*) downregulates their synthesis and secretion and promotes the production of their natural tissue inhibitors, TIMPs [11]. Gene and polymorphism identification could result in a new diagnostic for risk examination, early detection of disease, and individual treatment approach. Thus, genetic epidemiology includes knowledge about polymorphism genetic, which is promising as one of the tools that can contribute to the understanding of the periodontal disease. Gingival crevicular fluid could be a diagnostic tool for analysis of oral diseases. GCF, as a biomonitoring fluid, plays a constructive role in the diagnosis of oral diseases, especially for periodontitis and gingivitis. Its limited amount compromises biochemical and proteomic analysis, and the severity of inflammation in the periodontium affects its collection [12].

Gingival crevicular fluid is a serum exudate that originates from the periodontal sulcus or pocket and is regarded as a promising biological fluid for the detection of periodontal disease. Its composition resembles normal serum, but its volume fluctuates in certain conditions such as those of gingivitis, caries, external root resorption, and chronic periodontitis, as well as during orthodontic movement. GCF is composed of variable substances that include immunoglobulin, enzymes, local mediators, toxin cells, protein peptides, tissue breakdown products, and microorganisms [12].

The TGF-*β* superfamily consists of several multifunctional structurally related growth and differentiation factors associated with the inflammatory response. Five distinct isoforms of TGF-*β* have now been described, and three of these—TGF-*β*1, TGF-*β*2, and TGF-*β*3—are found in all mammalian species [11, 13]. TGF-*β*1 is expressed in epithelial, hematopoietic, and connective tissue cells. It is predominantly produced by T regulatory (Treg) cells and macrophages and could also induce a wide range of essential functions. Because TGF-*β*1 exhibits both proinflammatory and anti-inflammatory properties besides its ability to stimulate migration and synthesis of ECM molecules and to inhibit the breakdown of ECM, it has been intensively evaluated in relation to all types of gingival enlargement [11, 14].

Real-time polymerase chain reaction (RT-PCR) examination was conducted using TGF-*β*1 primary Macrogen to find TGF-*β*1 gene expression with results before gingivectomy and SRP treatment of 9.72121. A week after, gingivectomy and SRP treatment decreased to 4.10328, and three weeks after, gingivectomy and SRP treatment rebound to 9.7010. The expression of the TGF-*β*1 gene decreased on the seventh day after gingivectomy and SRP treatment and increased again on the 1st day, and the TGF-*β*1 gene here acted as an anti-inflammatory. Babel et al. who conducted a study involving patients with chronic periodontitis discovered that high TGF-*β*1 production might be a protective factor for periodontitis. Although TGF-*β*1 levels are elevated in moderate disease, they declined in fluid samples obtained from the pockets in more advanced experimental periodontitis [15].

Enzyme-linked immunosorbent assay (ELISA) examination was conducted to find TGF-*β*1 protein level using the Human TGF-*β*1 ELISA kit LSBio (Lifespan Biosciences Inc.) with results before gingivectomy and SRP treatment of 1129.736 pg/dl. A week after, gingivectomy and SRP treatment decreased to 662.242 pg/dl, and three weeks after, gingivectomy and SRP treatment rebound to 1079.391 pg/dl. These results were similar to those of the study conducted by Sattari et al. who found a significant decrease in TGF-*β*1 level from phase 1 (baseline or before surgery) to phase 3 (12 weeks after surgery). However, they did not assess the changes in TGF-*β*1 concentration between phase 1 and phase 2 (4 weeks after surgery) [14]. A study involving 60 patients by Mutlak et al. reported insignificant differences for the chronic periodontitis group in comparison with the control group, even though TGF-*β*1 depicted the highest correlation of the biochemical and immunohistological expression only in the chronic periodontitis group [16].

Gram staining has been done with bacteria in BHIB bacterial suspension, and gram-negative bacteria is obtained in the form of bacil composed of monobacil. The BHIB media are turbid indicating bacterial growth in the media. The bacteria are bacil and streptobacil. Bacteria in red are bacteria fading with alcohol but are able to bind to the dye comparator safranin. Positive results were found in all the sugars used (glucose, maltose, lactose, sucrose, and mannitol). Positive results are indicated by the color change indicator (from blue to yellow) contained in this medium.

The color change is caused by the bacteria that grow in it and are able to ferment all the confectionery products in the form of acid products. Positive results were obtained using Simmons' citrate agar because the color in the media is changed from green to blue. This is because the *Klebsiella* bacteria is one of the species that use citrate as a carbon source for metabolism by producing an alkaline atmosphere. In one series of urease biochemical tests, the results obtained are positive because the color of the media turns to pink.

Indole reaction can only be seen when this medium with growing bacteria is added with Covac's reagent. Indole is positive when it has a red ring on its surface. The red color is produced from the residual which results from the reaction of the amino acid tryptophan to indole with the addition of Covac's reagent. Bacteria capable of producing indole signify that the bacteria use the tryptophan amino acid as a carbon source. In the observation results obtained, indole was negative, so it can be concluded that the growing bacteria do not use tryptophan amino acids as the carbon source. From the result of identification and isolation that have been done (staining, breeding, differential test, biochemical test, and sugar) on the dental plaque sample, *Klebsiella* sp. bacteria were found before gingivectomy and SRP treatment.

The results of the identification of bacteria contained on plaque and calculus preparation through bacterial culture examination before gingivectomy and SRP treatment found the existence of *Klebsiella* sp.; then, on the first control, we did not find any *Klebsiella* sp. a week after gingivectomy and SRP treatment, but we found *Streptococcus* sp., and on the second control, three weeks after gingivectomy and SRP treatment, we still found *Streptococcus* sp.

A study conducted by Uzel et al. stated that *A. gerencseriae*, *A. israelii*, *A. odontolyticus*, *C. sputigena*, *E. nodatum*, *F. nucleatum* subsp. *polymorphum*, *F. nucleatum* subsp. *vincentii*, *F. periodonticum*, *P. nigrescens*, *T. denticola*, and *T. socranskii* were found in periodontally healthy subjects on day 1 observation. On the other hand, *C. rectus*, *E. nodatum*, *P. intermedia*, and *S. constellatus* were found in the periodontitis group. But *Veilonella parvula*, *Neisseria mucosa*, and *A. oris* were found in both groups. In this study, they compared the shift of microbes taken from the supragingival and subgingival plaque sample in healthy and periodontitis subjects before and after tooth cleaning. They also concluded that the hypothesis that biofilm redevelopment would be more rapid in periodontitis than in periodontally healthy subjects was rejected for supragingival biofilms but could not be rejected for subgingival biofilm redevelopment [17].

The result of anatomical pathology examination on gingival tissue macroscopically was that the tissue has a size of ±1 cm in diameter with red bright color while microscopically showed biopsy tissue was coated by epithelium squamosum complex which some seem hyperplastic but the nuclei within normal size, subephithelial composed of stroma of edematous fibrocollagenous tissues which was pounding with massive lymphosites, PMNs, and hystiocytes and were accompanied by vascular proliferation and hemorrhage, but there wasn't sign of malignancy. We concluded that this case was nonspecific gingival enlargement.

**Figure 2:**
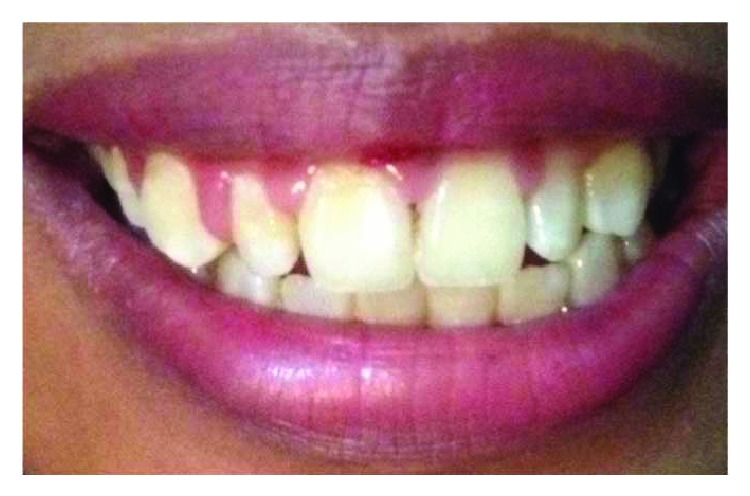
Clinical examination. An enlarged gingiva appears on the anterior and posterior teeth of the right maxilla region.

**Figure 3:**
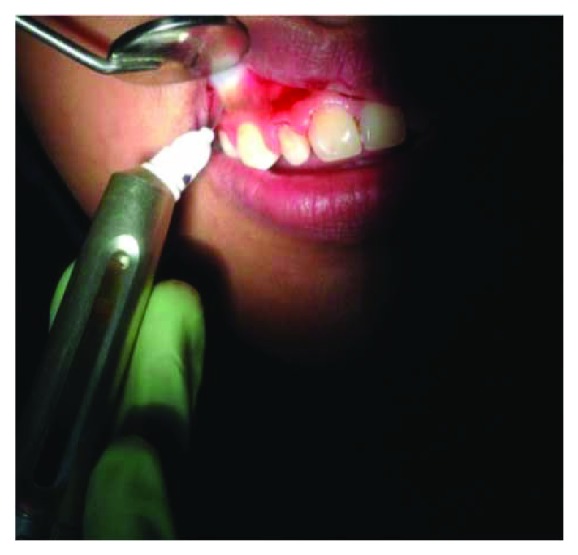
After disinfection, a local anesthetic was injected using 2% lidocaine norepinephrine.

**Figure 4:**
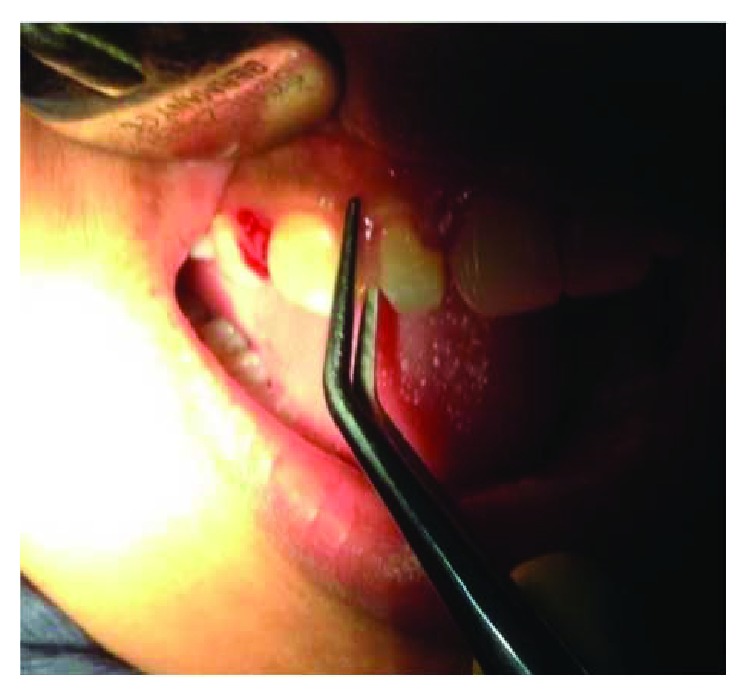
Determining the baseline pocket using an Ossung® pocket marker that will result in a bleeding point.

**Figure 5:**
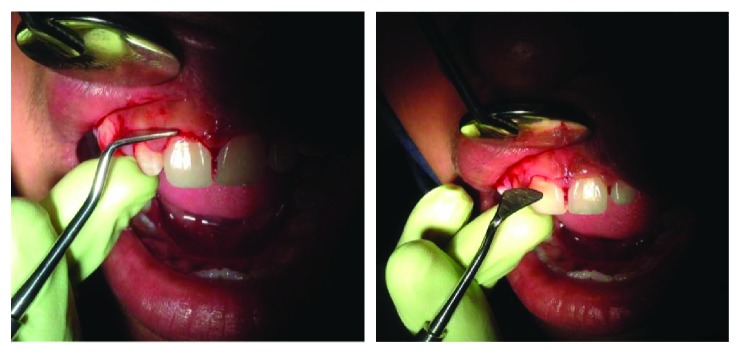
Incision and excision conducted on the buccal region of the gingiva using an Ossung Kirkland knife.

**Figure 6:**
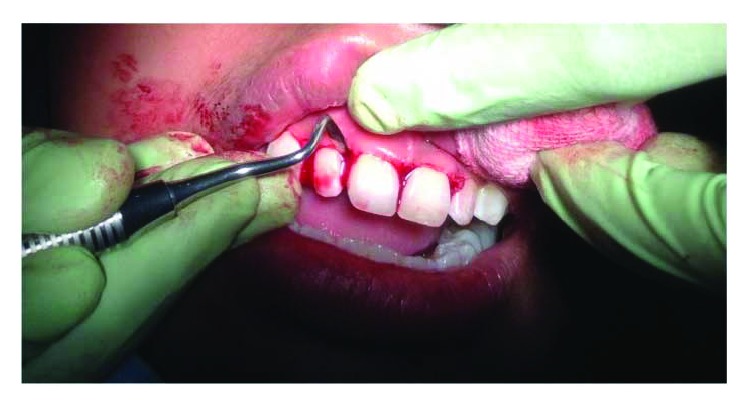
Incision and excision conducted on the interdental gingival area using an Ossung Orban knife.

**Figure 7:**
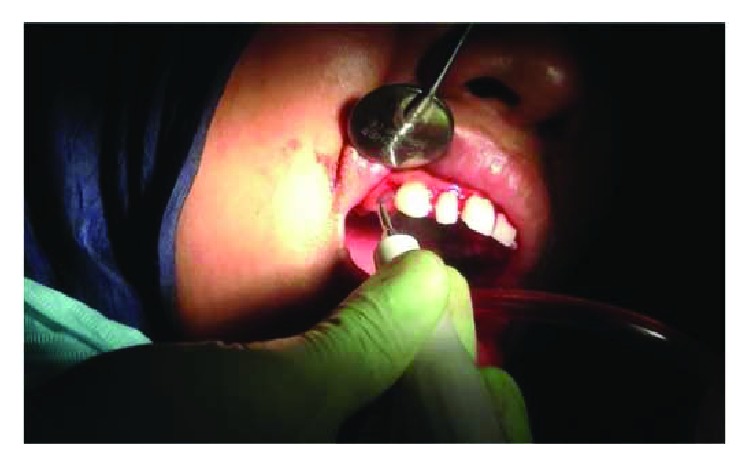
Scaling and root planing (SRP) performed with an electric scaler (Satelec P5 Newtron).

**Figure 8:**
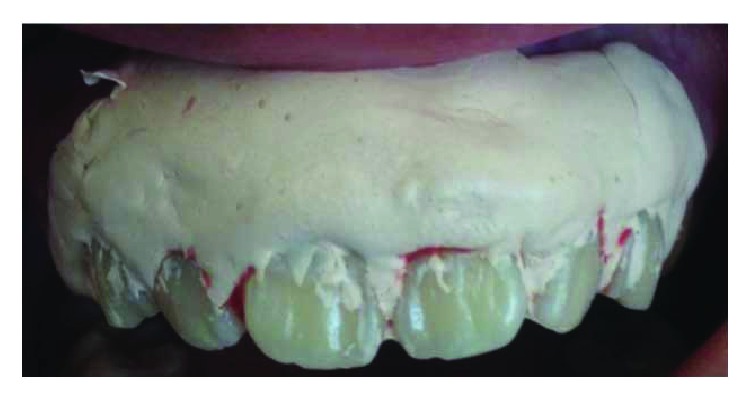
The periodontal pack being fixed after gingivectomy (Coe Pack®).

**Figure 9:**
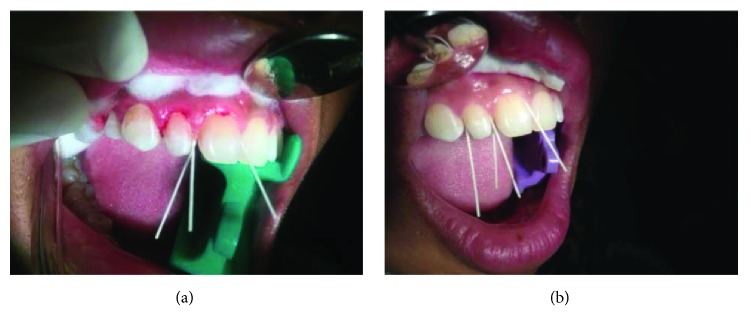
Gingival crevicular fluid (GCF) taken before gingivectomy (a) and after gingivectomy (b).

**Figure 10:**
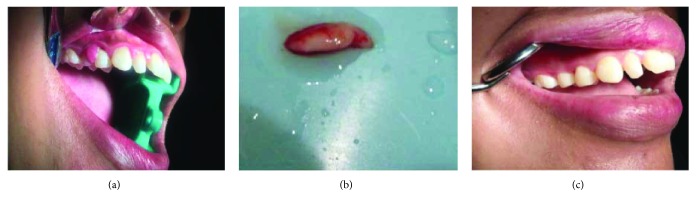
Appearance before gingivectomy (a), gingival tissue that has been excised (b), and appearance after gingivectomy (c).

On clinical examination (Figure 2), there are swollen gingivae in the anterior and posterior (labial, buccal) region of the right maxilla. The gingiva was seen to have edema and hyperemia on interdental 11, 12, 13, and 14. Bleeding occurred when the pocket depth (probing) was examined. The depth of the gingival pocket was approximately 4 mm in region 11, 12, 13, and 14. Besides, a plaque on the tooth surface and subgingival calculus was evident. There was no traumatic occlusion in the maxillary anterior teeth and mandibular anterior teeth. On the other hand, there is no tooth mobility found. Povidone iodine was used for disinfection; then, local anesthetic infiltration was performed using 2% lidocaine mixed with norepinephrine in the labial and buccal part of the tooth 11, 12, 13, and 14 region (Figure 3). Furthermore, the pocket base was marked using a pocket marker to obtain the bleeding point on each enlarged gingiva. This procedure was performed to obtain the pocket base as a reference for gingivectomy (Figure 4). Gingival incision was performed on the bleeding point at the buccal region using a Kirkland knife. It was placed on the enlarged interdental area of the gingiva of the enlarged teeth. The Kirkland knife was placed at 45° to the gingiva to obtain a bevel on the gingival surface (Figure 5). Gingival excision on the interdental part of the pocket base was performed to take the gingival tissue that has been enlarged due to inflammation using an Orban knife. Furthermore, scaling and root planing was performed. Gingival tissue removal can be done after the previous incision (Figure 6). Scaling and root planing is performed to eliminate plaque and calculus, especially in subgingival areas using an electric scaler. Irrigation was performed using a 0.12 chlorhexidine solution in areas where gingivectomy and scaling have been performed. This is to make sure that the area is clean from plaque and calculus (Figure 7). The final procedure is the fastening of periodontal dressing using periodontal pack that covers all gingival areas where gingivectomy has been performed. The utilization of periodontal dressing is to ease the healing process. Placement of periodontal dressing does not cover the entire surface of the tooth for aesthetic reason (Figure 8). GCF has been taken from the gingival sulcus before the gingivectomy procedure using size 15 paper points (Figure 9(a)). They are placed on the interdental and buccal areas of teeth 11 and 12 (Figure 9(b)). The clinical features before gingivectomy are shown in Figure 10(a). The gingival tissue that has undergone gingivectomy is shown in Figure 10(b). As shown in Figure 10(c), a change of contour on the gingival surface was observed 3 weeks after gingivectomy, no reenlargement and no bleeding were observed, and edema, hyperemia, and attached gingiva formed well on the tooth surface.

## Figures and Tables

**Table 1:** Change of TGF-*β*1 gene expression and TGF-*β*1 protein level in gingival crevicular fluid (GCF).

	Before gingivectomy (day 1)	After gingivectomy (day 7)	After gingivectomy (day 21)
Change of TGF-*β*1 gene expression	9.72121	4.10328	9.7010
Change of TGF-*β*1 protein level	1129.736 pg/dl	662.242 pg/dl	1079.391 pg/dl

**Table 2:** Identification of plaque bacteria.

Before gingivectomy (day 1)	After gingivectomy (day 7)	After gingivectomy (day 21)
*Klebsiella* sp.	*Streptococcus* sp.	*Streptococcus* sp.
